# Growth performance and carcass traits of two commercial broiler strains fed diet supplemented with essential oils

**DOI:** 10.1016/j.heliyon.2022.e12094

**Published:** 2022-12-02

**Authors:** Mohammad D. Obeidat, Basheer M. Nusairat, Belal S. Obeidat

**Affiliations:** Department of Animal Production, Faculty of Agriculture, Jordan University of Science and Technology (JUST), Irbid 22110, Jordan

**Keywords:** Strain, Essential oils, Growth, Carcass

## Abstract

The current study aimed to evaluate the efficacy of using essential oils (EOs) on growth, carcass, and meat quality traits of two commercial broiler strains raised to 35 days of age. A total of 384 chicks were obtained upon hatching from a local hatchery (192 Indian River and 192 Hubbard). Birds were allocated randomly according to their strain into three groups: control, EOs, and EOs grower. Body weight was recorded at the beginning of the trial and then at the end of each phase diet as well as for the feed intake. Sixteen birds from the combination of each strain-essential oil were chosen randomly to evaluate carcass characteristics at the end. Hubbard consumed more feed during the grower stage (p = 0.02) and overall (p = 0.002) compared to Indian River. Carcass cuts percentages were affected by strain (p < 0.01). Shear force was lower for the Hubbard (p = 0.002). Essential oils showed a significant effect on cooking loss (p = 0.03). A significant strain by essential oil interactions was obtained for cooking loss, shear force, and meat redness (color coordinate ∗a) Cooking loss was greater for the Indian river with the EOs grower treatment. Briefly, growth and carcass traits were affected by strain. The inclusion of EOs had slightly improved meat quality traits.

## 1 Introduction

The production of healthy chicken requires proper microbial control (Ravindran, 2013). In the past decades antibiotics were used in broiler diets mainly to control microbial infections, however, due to the concern of using these compounds and their adverse effect on both humans and the environment, they have been banned in many countries around the world (Demir et al., 2005). Antibiotics residues and antibiotic resistant bacteria strains have directed the researchers to find antibiotic alternatives in poultry diet (Ruff et al., 2021) Different types of feed additives such as probiotics, prebiotics, synbiotics, organic acids, enzymes, and phytogenic were used in poultry diets to supplant antibiotics (Gibson et al., 2004; Musa et al., 2009; Bedford and Cowieson 2012; Dhama et al., 2015; Upadhaya et al. 2016). The use of essential oils in poultry diets has received a lot of attention as a potential alternative to antibiotic growth promoters at sub-therapeutic doses, which limits bacterial growth to improve gut health and consequently growth performance (Cherian et al., 2013; Stefanello et al., 2020). Increasing nutrient digestibility, and digestive enzymes action, as well as antimicrobial activity, are the main advantages of phytogenic compounds (Hernández et al., 2004; Peri et al., 2009). In addition, the effect of lipid oxidation on broiler's meat quality and fatty acid content during the storage period is another important thing for the producers (RHEE et al., 2006). For this reason, a dietary source of antioxidants was used to enhance meat stability (Giannenas et al., 2016). Essential oils as one of the phytogenic compound are known to show antioxidants abilities (Windisch et al., 2008), and had shown positive influence on the stability of fatty substance to degradation processes (Bobko et al., 2016) and thus can be used to reduce lipid oxidation.

Essential oils could diminish microbial threats and improve intestinal health and thus growth performance of broilers (Bortoluzzi et al., 2021), which is considered a very important factor that is responsible for the best bird performance and profit. The goal of this study was to evaluate the efficacy of essential oils as an alternative to antibiotic growth promoters in terms of growth performance, carcass traits, and meat quality in the two main commercial broiler strains used in Jordan.

## 2 Materials and methods

### 2.1 Experimental birds and housing

**Figure 1:**
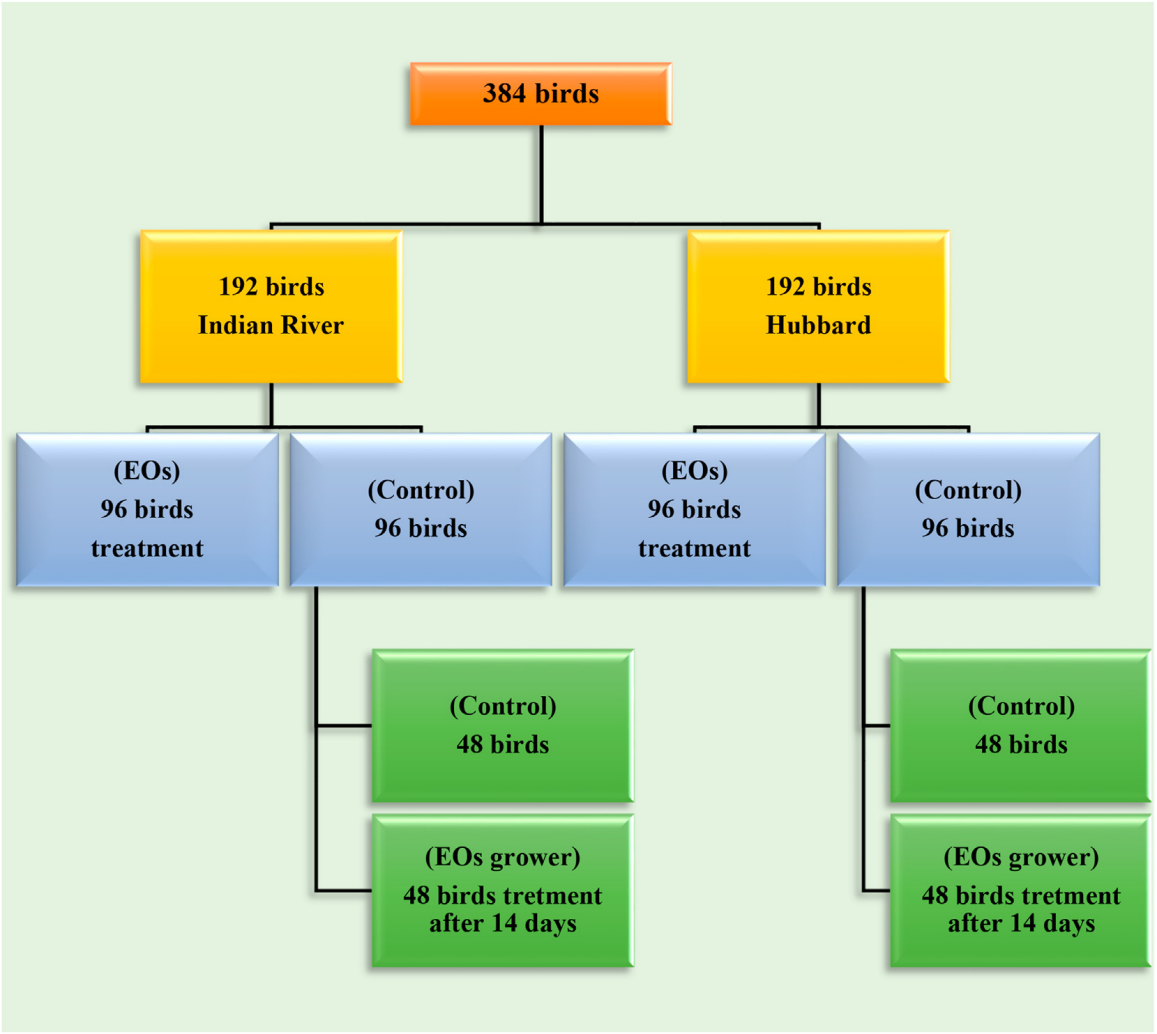
Experimental design illustration: per strain; birds were receiving either control (16 replicate cages) or EOs (8 replicate cages) from 0-14 d. From 15-35 d, pre-assigned 8 replicate cages from control group received essential oil blend (EOs grower) at inclusion rate of 125 g/ton.

The experiment was conducted in the Animal House at Jordan University of Science and Technology (JUST) during spring 2017. All animal care protocols and experimental procedures were approved by the Animal Care and Use Committee at JUST (Approval #: 20/5/4/136). Birds were kept in cages in a climate-controlled room (temperature (22 °C), humidity (50%)) and provided free access to water and feed (mash form). A total of 384 one-day-old chicks were obtained from a local hatchery (192 Indian River and 192 Hubbard). At placement, birds were randomly allocated to 24 cages per strain with 12 birds per replicate, 16 cages received no additive (Control group), while the remaining 8 cages received essential oil blend (Digestarom®, Biomin, Austria) in feed (EOs group) at inclusion rate of 125 g/ton up to 14 days of age. On day 15, 8 cages that were consuming control diet (pre-assigned as EOs grower group) were fed essential oil from 15-35 days of age at an inclusion rate similar to EOs group (Figure 1). Digestarom® is a phytogenic feed additive that is composed of a blend of essential oils (peppermint, eugenol or clove, anise, and thyme). Birds were challenged by reducing ration’s crude protein content for both starter and grower phases by 1.5%, and metabolizable energy (ME) in both starter and grower by 75 kcal/kg (Table 1).

**Table 1:** Feed formulation.

Ingredients %	Starter^1^ 0–14 d	Grower^2^ 15–35 d
Corn	54.18	56.07
SBM	38.37	35.20
Soy oil	2.08	3.50
Limestone	1.44	1.40
Monocalcium Phosphate	1.70	1.70
Vitamin and mineral premix^3^	0.01	0.01
Salt	0.50	0.50
DL-Methionine	0.29	0.25
Choline Chloride	0.20	0.20
L-Lysine	0.14	0.08
L-Threonine	0.04	0.00
Sand^4^	1.05	1.09
**Calculated nutrients**		
Protein %	21.5	19.5
Dig. Lysine %	1.22	1.07
Dig. (Methionine + Cystine)%	0.90	0.81
Calcium%	0.94	0.91
Available P%	0.48	0.47
ME, Kcal/kg	2925	2975

1 From 0 to 14 days of age, two treatments (Control and control plus EO).

2 From 15 to 35 days of age control was divided into two groups, the first group remained control while the other received EOs (EOs grower).

3 Vitamin and trace mineral premix supplied the following per kg of diet: 5,512 IU vitamin A, 1,852 IU vitamin D3, 11 IU vitamin E, 0.06 mg vitamin B12, 0.23 mg biotin, 1.87 mg menadione (K3), 0.44 mg thiamine, 3.75 mg riboflavin, 5.95 mg d-pantothenic acid, 1.32 mg vitamin B6, 34.17 mg niacin and 0.22 mg folic acid, for mineral supplied the following per kg of diet: Manganese: 120 mg, Zinc: 120 mg, Iron: 80 mg, Copper: 10 mg, Iodine, 2.5 mg, Cobalt, 1 mg.

4 Sand used as inert filler to include Digestarom®.

### 2.2 Growth performance and slaughtering procedures

Birds from each group were weighed at the beginning of the study and weekly thereafter. Birds were checked daily for all groups throughout the duration of the experiment. Feed refusals were recorded weekly to calculate feed intake (FI). Feed conversion ratio (FCR) adjusted for mortality was determined as the ratio between total FI to final body weight gain (BWG). On day 35 final live weight of the birds was recorded after being fasted for 12 h, then sixteen birds from each treatment were randomly selected and slaughtered in accordance with the protocol outlined by (Mahmoud et al., 2015) by cutting the carotid arteries and jugular vein using a knife. Carcasses were then washed and chilled at 4 °C in clean water for 30 min. Carcasses were weighed and then divided into 5 cuts (breast, legs, wings, neck, back, and abdominal fat pad (AFP) surrounding the bursa of Fabricius and cloaca). All cuts weights were obtained and dressing percentage was calculated as the ratio of carcass weight to live weight.

### 2.3 Meat quality traits

All meat quality traits were measured on right and left pectoralis muscle of the broiler breast. Traits included cooking loss (CL), shear force (SF), water holding capacity (WHC), pH, and color coordinates. Cooking loss was (CL) expressed as the weight lost during cooking for 25 min at 85 °C in water bath divided by the fresh sample weight of the pectoralis muscle and expressed as a percentage, Warner-Bratzler shear force (SF) reported as the average of the maximum force of the four replicates from each pectoralis major muscle sample using (Warner-Bratzler meat shear, G-R manufacturing co. 1317 Collins LN, Manhattan, Kansas, 66502, USA). Color coordinates (yellowness, b; whiteness, l; redness, a) following the terms of CIELAB (Commission International de l’Eclairage, 1976), pH using (pH spear, large screen, waterproof pH/temperature tester, double injection, model 35634–40, Eurotech Instruments, Malaysia), and water holding capacity (WHC) of the pectoralis muscle assessed by measuring the quantity of water extracted from the muscle protein by applying pressure (expressible juice) and the capability of muscle protein to retain water present in excess and under the impact of internal force. All procedures for quality traits were followed as described by (Mahmoud et al., 2015).

### 2.4 Statistical analysis

Data collected were analyzed using the PROC GLM procedure of SAS (SAS Inst., Inc., Cary, NC). For growth performance traits, models included strain, feed additive treatment, and their interactions as fixed effects.

(1) $$Y_{ijk\;}=\mu +\alpha_{i\;}+\beta_{j+\alpha }\beta_{ij}\;{+Ɛ}_{ijk}$$

Where:

Y_ij_ the dependent variable

μ is the overall mean

α_i_ is the strain effect, where i = Hubbard or Indian River

β_j_ is the feed additive effect, where j = control, B, and C

αβ_ij_ the strain feed additive interaction.

Ɛ_ijk_ is the random error.

Level of significance was determined at alpha 0.05, the Tukey-Kramer mean separation test was used to determine differences between means.

For carcass and meat quality traits, models included strain, feed additive treatment, and their interactions as fixed effects, whereas carcass weight was included as covariate.

(2) $$Y_{ijk\;}=\mu +\alpha_{i\;}+\beta_{j+\alpha }\beta_{ij+}bCW+{Ɛ}_{ijk}$$

Where:

Y_ij_ the dependent variable

μ is the overall mean

α_i_ is the strain effect, where i = Hubbard or Indian River.

β_j_ is the feed additive effect, where j = control, B, and C

αβ_ij_ the strain feed additive interaction

ƅCW linear covariate of carcass weight.

Ɛ_ijk_ is the random error.

Level of significance was determined at alpha 0.05, the Tukey-Kramer mean separation test was used to determine differences between means.

## 3 Results and discussion

### 3.1 Growth performance

#### 3.1.1 Feed intake

Results of feed intake are presented in Table 2. During the first two weeks of age (starter phase), feed intake for Hubbard and Indian River did not significantly differ. On the contrary, overall feed consumption and during grower period was higher (*p < 0*.05) for Hubbard compared to their Indian River counterparts. Similarly, strain was found to have a significant impact on birds feed consumption (Kalia et al., 2017). In the current study, there were no significant differences between the experimental diet regarding feed intake. Similarly, dietary supplementation of various levels of Digestarom have shown no effect on bird’s feed intake (Kalia et al., 2017). In addition, these results are in agreement with (Sadek et al., 2014), who found that adding a blend of EOs and herbal plants to broiler’s diet did not affect feed intake. In the current study, there was no significant strain-treatment interaction with regards to feed intake.

**Table 2:** Least square means for feed intake (g) of broilers as affected by strain^1^ and essential oils (EOs)^2^.

		Age (days)		
		1–14	15–35	1–35
**Strain**	**Hubbard**	481.43 ± 22.47	2731.62 ± 39.78	3244.34 ± 46.92
	**Indian River**	358.61 ± 12.30	2437.04 ± 37.09	2781.41 ± 40.75
	***P-value***	***NS***	***0.02***	***0.002***
**Treatment**	**Control**	396.28 ± 34.58	2560.91 ± 62.52	2963.91 ± 90.06
	**EOs**	431.14 ± 25.51	2617.85 ± 55.25	3050.72 ± 68.72
	**EOs grower**	-	2574.24 ± 52.92	3024.00 ± 82.99
	***P-value***	***NS***	***NS***	***NS***
**Interaction**				
**Control**	**Hubbard**	470.01 ± 46.60	2713.95 ± 106.51	3188.19 ± 120.81
**Control**	**Indian**	354.72 ± 45.49	2407.87 ± 93.13	2739.63 ± 105.63
**EOs**	**Hubbard**	499.32 ± 34.39	2758.02 ± 75.38	3273.08 ± 85.49
**EOs**	**Indian**	362.96 ± 33.00	2477.68 ± 72.36	2828.36 ± 82.06
**EOs grower**	**Hubbard**	-	2722.90 ± 100.57	3271.74 ± 114.06
**EOs grower**	**Indian**	-	2425.57 ± 103.09	2776.25 ± 116.93
	***P-value***	***NS***	***NS***	***NS***

^"NS" abbreviation refers to "non-significant" difference^.

1 Values are LSMeans ± SEM; n = 32.

2 Values are LSMeans ± SEM; n = 16 replicates (12 birds each) for control and EOs treatments each up to 14 days of age. At 15 days of age, 8 replicates (96 birds) from control were randomly assigned to EOs grower treatment: n = 8, 16, and 8 for control, EOs and EOs grower treatments, respectively.

### 3.2 Body weight gain

Hubbard had higher (*p < 0*.05) initial weight compared to Indian River birds. Final body weight for Hubbard strain was 137 g higher (*p = 0*.05) compared to Indian River. Birds from the Hubbard strain tended (*p = 0*.06) to have more overall weight gain than Indian River strain, 1922 vs 1785 g. No strain significant effect was found in BWG during starter or grower periods. The formulated ration had reduced protein % and ME content compared to the strain guideline recommendations, and thus, probably strains were not able to express their genetic potential for BWG. In the current study, diet effect was not significant for either body weight or body weight gain. Also, Pirgozliev et al., 2019 reported that BWG was not affected by EOs inclusion to the diet of Ross 308. Essential oils were found to improve nutrient digestibility and eventually improve growth performance of the birds, probably the low inclusion rate in the current study could not make a difference in nutrient digestibility and thus could not improve bird’s growth performance. In addition, the interaction did significantly not affect the weight traits as shown in Table 3.

**Table 3:** Least square means for body weight, and body weight gain (BWG) of broilers as affected by strain^1^ and essential oils (EOs)^2^.

		Initial BW (g)	BWG (g)			Final BW (g)
			Age (d)			
			1–14	15–35	1–35	
**Strain**	**Hubbard**	47.35 ± 0.27	358.41 ± 4.75	1563.96 ± 18.01	1922.32 ± 18.83	1966.62 ± 39.47
	**Indian River**	41.57 ± 0.27	334.78 ± 9.20	1450.25 ± 24.45	1785.00 ± 23.43	1829.29 ± 36.96
	***P-value***	***0.001***	***NS***	***NS***	***0.06***	***0.052***
**Treatment**	**Control**	44.80 ± 0.37	340.50 ± 10.05	1494.64 ± 44.47	1835.05 ± 44.11	1879.34 ± 32.71
	**EOs**	44.38 ± 0.26	350.87 ± 9.74	1509.58 ± 20.66	1860.50 ± 20.82	1904.80 ± 21.69
	**EOs grower**	-	-	1517.09 ± 27.02	1865.44 ± 30.56	1909.72 ± 29.19
	***P-value***	***NS***	***NS***	***NS***	***NS***	***NS***
**Interaction**						
**Control**	**Hubbard**	47.60 ± 0.52	357.56 ± 20.01	1549.82 ± 58.98	1911.17 ± 57.92	1955.37 ± 57.88
**Control**	**Indian**	42.00 ± 0.52	332.90 ± 18.5	1439.46 ± 51.58	1758.92 ± 50.65	1803.30 ± 50.61
**EOs**	**Hubbard**	46.84 ± 0.37	359.19 ± 14.54	1535.15 ± 41.75	1894.49 ± 40.99	1938.81 ± 40.96
**EOs**	**Indian**	41.92 ± 0.37	342.56 ± 13.96	1484.01 ± 40.06	1826.50 ± 39.34	1870.79 ± 39.31
**EOs grower**	**Hubbard**	47.63 ± 0.52	-	1606.90 ± 55.69	1961.28 ± 54.69	2005.67 ± 54.65
**EOs grower**	**Indian**	40.79 ± 0.52	-	1427.29 ± 57.09	1769.60 ± 56.06	1814.77 ± 56.02
	***P-value***	***NS***	***NS***	***NS***	***NS***	***NS***

^"NS" abbreviation refers to "non-significant" difference^.

1 Each strain had 32 replicates.

2 Values are LSMeans ± SEM; n = 16 replicates (12 birds each) for control and EOs treatments each up to 14 days of age. At 15 days of age, 8 replicates (96 birds) from control were randomly assigned to EOs grower treatment: n = 8, 16, and 8 for control, EOs and EOs grower treatments, respectively.

### 3.3 Feed conversion ratio (FCR)

Adjusted feed conversion ratio results are presented in Table 4. Even with the greater feed consumption of the Hubbard birds during finisher period and overall, strain did not significantly affect the values of FCR. Likewise, overall FCR ratio was comparable in both Ross 308 and Cobb 500 (Hristakieva et al., 2014). In addition, diet and diet-strain interaction did not show significant effect on FCR. Previous works showed that dietary supplementation of different levels of EOs had no effect on feed bird’s FCR for Vencobb, RIR crossbred, and hubbard (Kalia et al., 2017) and Cobb 500 (Mountzouris et al., 2011). On the contrary, adding Digestarom to Cobb 500 broiler’s feed has improved FCR compared to control diet (Sadek et al., 2014). In the current study, the inclusion rate of Digestarom was 0.0125% while in (Sadek et al., 2014) it was 1.5–3% of the diet, this difference could be responsible for difference in the results.

**Table 4:** Least square means for adjusted feed conversion ratio (AdjFCR) of broilers as affected by strain^1^ and essential oils (EOs)^2^.

		Age (days)		
		1–14	15–35	1–35
**Strain**	**Hubbard**	1.341 ± 0.06	1.759 ± 0.04	1.706 ± 0.03
	**Indian River**	1.070 ± 0.04	1.689 ± 0.03	1.553 ± 0.03
	***P-value***	***NS***	***NS***	***NS***
**Treatment**	**Control**	1.154 ± 0.09	1.732 ± 0.07	1.634 ± 0.06
	**EOs**	1.229 ± 0.07	1.739 ± 0.04	1.638 ± 0.03
	**EOs grower**	-	1.700 ± 0.03	1.617 ± 0.04
	***P-value***	***NS***	***NS***	***NS***
**Interaction**				
**Control**	**Hubbard**	1.351 ± 0.13	1.768 ± 0.09	1.706 ± 0.08
**Control**	**Indian**	1.071 ± 0.12	1.696 ± 0.08	1.561 ± 0.07
**EOs**	**Hubbard**	1.389 ± 0.10	1.807 ± 0.06	1.738 ± 0.06
**EOs**	**Indian**	1.069 ± 0.09	1.670 ± 0.06	1.537 ± 0.05
**EOs grower**	**Hubbard**	-	1.701 ± 0.09	1.673 ± 0.07
**EOs grower**	**Indian**	-	1.699 ± 0.09	1.561 ± 0.08
	***P-value***	***NS***	***NS***	***NS***

^"NS" abbreviation refers to "non-significant" difference^.

1 Each strain had 32 replicates.

2 Values are LSMeans ± SEM; n = 16 replicates (12 birds each) for control and EOs treatments each up to 14 days of age. At 15 days of age, 8 replicates (96 birds) from control were randomly assigned to EOs grower treatment: n = 8, 16, and 8 for control, EOs and EOs grower treatments, respectively.

### 3.4 Carcass and meat quality traits

#### 3.4.1 Carcass traits

Carcass traits are presented in Table 5. Hubbard strain tended (*p = 0*.07) to have more carcasses weight compared with the Indian River strain. Legs, wings, back, and (AFP) percentages were higher (*p <* 0.01) for Hubbard birds compared to Indian River. However, breast cut percentage was higher (*p <* 0.01) for the Indian River strain. In parallel, breast, leg, and AFP percentages were affected by broiler strain, while dressing percentage was not affected (Abdullah et al., 2010; Benyi et al., 2015). Moreover, the EO inclusion showed no significant effect on carcass traits. Comparably, adding PFA (Digestarom®) did not affect dressing percentage, breast, and thigh in broiler carcasses (Syed, 2019). In addition, slaughter weight, carcass, dressing percentage, and AFP percentages were not affected by PFA (Abd El-Hady et al., 2020). No strain-treatment interaction was found for carcass traits.

**Table 5:** Least square means for carcass traits of broilers as affected by strain^1^ and essential oils (EOs)^2^.

		Traits								
		Slaughter weight (g)	CCWT (g) 3	DP%^4^	Brest %	Legs %	Wings %	Back %	Neck %	AFP^5^ %
**Strain**	**Hubbard**	1905.83 ± 21.9	1462.4 ± 15.0	77.5 ± 0.86	33.4 ± 0.3	27.9 ± 0.2	10.1 ± 0.1	18.9 ± 0.1	9.4 ± 0.9	1.2 ± 0.05
	**Indian River**	1849.79 ± 21.9	1431.0 ± 15.0	76.9 ± 0.86	36.0 ± 0.3	27.1 ± 0.2	9.7 ± 0.1	18.1 ± 0.1	7.5 ± 0.9	0.8 ± 0.05
	***P-value***	***NS***	***NS***	***NS***	***< 0.01***	***0.001***	***0.0001***	***0.0001***	***NS***	***0.001***
**Treatment**	**Control**	1863.4 ± 26.88	1426.1 ± 18.3	76.3 ± 1.1	34.4 ± 0.3	27.7 ± 0.2	9.9 ± 0.1	18.7 ± 0.1	9.4 ± 1.1	0.99 ± 0.06
	**EOs**	1885.9 ± 26.88	1449.3 ± 18.3	77.2 ± 1.1	35.0 ± 0.3	27.1 ± 0.2	10.0 ± 0.1	18.3 ± 0.1	7.8 ± 1.1	1.0 ± 0.06
	**EOs grower**	1884.1 ± 26.88	1464.8 ± 18.3	78.1 ± 1.1	34.7 ± 0.3	27.6 ± 0.2	9.9 ± 0.1	18.4 ± 0.1	8.0 ± 1.1	0.98 ± 0.06
	***P-value***	***NS***	***NS***	***NS***	***NS***	***NS***	***NS***	***NS***	***NS***	***NS***
**Interactions**										
**Control**	**Hubbard**	1906.9 ± 38.0	1434.3 ± 25.9	76.1 ± 1.5	32.8 ± 0.4	28.4 ± 0.3	10.0 ± 0.1	19.1 ± 0.2	11.6 ± 1.6	1.2 ± 0.09
**Control**	**Indian**	1820 ± 38.0	1417.8 ± 26.1	76.6 ± 1.5	35.9 ± 0.4	27.0 ± 0.3	9.8 ± 0.1	18.2 ± 0.2	7.2 ± 1.6	0.8 ± 0.09
**EOs**	**Hubbard**	1870 ± 38.0	1446.8 ± 25.8	77.2 ± 1.5	33.8 ± 0.4	27.3 ± 0.3	10.2 ± 0.1	18.7 ± 0.2	8.0 ± 1.6	1.2 ± 0.09
**EOs**	**Indian**	1901.9 ± 38.0	1451.7 ± 25.9	77.3 ± 1.5	36.2 ± 0.4	26.9 ± 0.3	9.7 ± 0.1	18.0 ± 0.2	7.7 ± 1.6	0.8 ± 0.09
**EOs grower**	**Hubbard**	1940.6 ± 38.0	1506.1 ± 26.2	79.3 ± 1.5	33.5 ± 0.4	27.9 ± 0.3	10.1 ± 0.1	18.8 ± 0.2	8.4 ± 1.6	1.1 ± 0.09
**EOs grower**	**Indian**	1827.5 ± 38.0	1423.6 ± 26.1	76.8 ± 1.5	35.9 ± 0.4	27.4 ± 0.3	9.7 ± 0.1	18.0 ± 0.2	7.6 ± 1.6	0.8 ± 0.09
	***P-value***	***NS***	***NS***	***NS***	***NS***	***NS***	***NS***	***NS***	***NS***	***NS***

^"NS" abbreviation refers to "non-significant" difference^.

1 Each strain had 32 replicates.

2 Values are LSMeans ± SEM; n = 16 replicates (12 birds each) for control and EOs treatments each up to 14 days of age. At 15 days of age, 8 replicates (96 birds) from control were randomly assigned to EOs grower treatment. For carcass traits n = 16 for control, EOs and EOs grower treatments, respectively.

3 Cold Carcass Weight.

4 Dressing Percentage.

5 AFP is the abdominal fat pad.

#### 3.4.2 Meat quality traits

The breast fillet percentage was higher (*p <* 0.01) for the Indian River than Hubbard, 36 and 33%, respectively (Table 6). Hubbard birds showed more WHC (*p = 0*.03) which has resulted in lower shear force (*p = 0*.002) compared to Indian River. Both (L and b) color coordinates values were greater (*p <* 0.01) for the Indian River birds. Likewise, strain of the chicken (Ninghai chicken, frizzle chicken, Ninghai xiang chicken, and Zhenning loquat chicken, and one genotype of commercial broiler Arbor Acres plus broiler) was found to have a significant influence on shear force and color coordinates (Guan et al., 2013). Highly significant negative correlation (r = -0.27, *p* = 0.006) between lightness color coordinate (L) and abdominal fat pad was observed, and thought to be responsible for the lower (L) value for the Hubbard birds as their abdominal fat pad was greater than Indian River. Adding EOs showed a significant effect on cooking loss (*p = 0*.03), were birds received EOs treatment group had lower cooking loss compared to the control group. However, all other traits were not significantly affected by EOs inclusion. Similarly, the inclusion of EOs did not affect meat pH, WHC, and color coordinates of broiler chickens (Li et al., 2015), while, they have caused a reduction in cooking loss in quail meat (Hazrati et al., 2020; Abdel-Wareth et al., 2022).

**Table 6:** Least square means for meat quality traits of broilers as affected by strain^1^ and essential oils (EOs)^2^.

		Traits							
		Quality measures					Color coordinates^3^		
		Breast Fillet %	pH	CL%	WHC%	SF	l	a	b
**Strain**	**Hubbard**	69.1 ± 0.9	5.9 ± 0.01	27.4 ± 0.5	30.6 ± 0.7	2.6 ± 0.1	41.8 ± 03	2.9 ± 0.04	18.3 ± 0.1
	**Indian River**	73.1 ± 0.9	5.9 ± 0.01	26.4 ± 0.5	28.5 ± 0.7	3.0 ± 0.1	44.4 ± 0.3	2.9 ± 0.0.4	18.9 ± 0.1
	***P-value***	***0.003***	***NS***	***NS***	***0.03***	***0.002***	***0.001***	***NS***	***0.004***
**Treatment**	**Control**	71.5 ± 1.1	5.9 ± 0.02	28.0 ± 0.6^a^	30.0 ± 0.9	2.9 ± 0.1	42.8 ± 0.3	2.9 ± 0.05	18.5 ± 0.2
	**EOs**	70.8 ± 1.1	5.9 ± 0.02	25.6 ± 0.6^b^	28.6 ± 0.9	2.8 ± 0.1	42.9 ± 0.3	2.8 ± 0.05	18.7 ± 0.2
	**EOs grower**	71.1 ± 1.1	5.8 ± 0.02	27.1 ± 0.6^ab^	30.1 ± 0.9	2.8 ± 0.1	43.5 ± 0.3	2.8 ± 0.05	18.5 ± 0.2
	***P-value***	***NS***	***NS***	***0.03***	***NS***	***NS***	***NS***	***NS***	***NS***
**Interaction**									
**Control**	**Hubbard**	69.1 ± 1.6	5.9 ± 0.03	29.3 ± 0.9^a^	30.1 ± 1.2	2.4 ± 0.2^c^	41.5 ± 0.4	3.0 ± 0.07^a^	18.4 ± 0.2
**Control**	**Indian**	73.8 ± 1.6	5.9 ± 0.03	26.6 ± 0.9^ab^	29.9 ± 1.2	3.3 ± 0.2^a^	44.2 ± 0.4	2.9 ± 0.07^ab^	18.7 ± 0.2
**EOs**	**Hubbard**	68.9 ± 1.6	5.9 ± 0.03	26.9 ± 0.9^bc^	29.9 ± 1.2	2.4 ± 0.2^c^	41.7 ± 0.4	2.9 ± 0.07^a^	18.6 ± 0.2
**EOs**	**Indian**	72.7 ± 1.6	5.9 ± 0.03	24.4 ± 0.9^bc^	27.3 ± 1.2	3.2 ± 0.2^a^	44.1 ± 0.4	2.6 ± 0.07^c^	18.9 ± 0.2
**EOs grower**	**Hubbard**	69.5 ± 1.6	5.9 ± 0.03	26.0 ± 0.9^b^	31.8 ± 1.2	3.0 ± 0.2^ab^	42.2 ± 0.4	2.7 ± 0.07^bc^	17.9 ± 0.2
**EOs grower**	**Indian**	72.7 ± 1.6	5.8 ± 0.03	28.2 ± 0.9^ac^	28.3 ± 1.2	2.7 ± 0.2^bc^	44.8 ± 0.4	3.0 ± 0.07^a^	19.1 ± 0.2
	***P-value***	***NS***	***NS***	***0.01***	***NS***	***0.002***	***NS***	***0.005***	***NS***

Mean values within a column for each variable with different superscripts differ significantly (p < .05).

^"NS" abbreviation refers to "non-significant" difference^.

1 Each strain had 32 replicates.

2 Values are LSMeans ± SEM; n = 16 replicates (12 birds each) for control and EOs treatments each up to 14 days of age. At 15 days of age, 8 replicates (96 birds) from control were randomly assigned to EOs grower treatment. For carcass traits n = 16 for control, EOs and EOs grower treatments, respectively.

3 l = whiteness, b = yellowness, a = redness.

**Figure 2:**
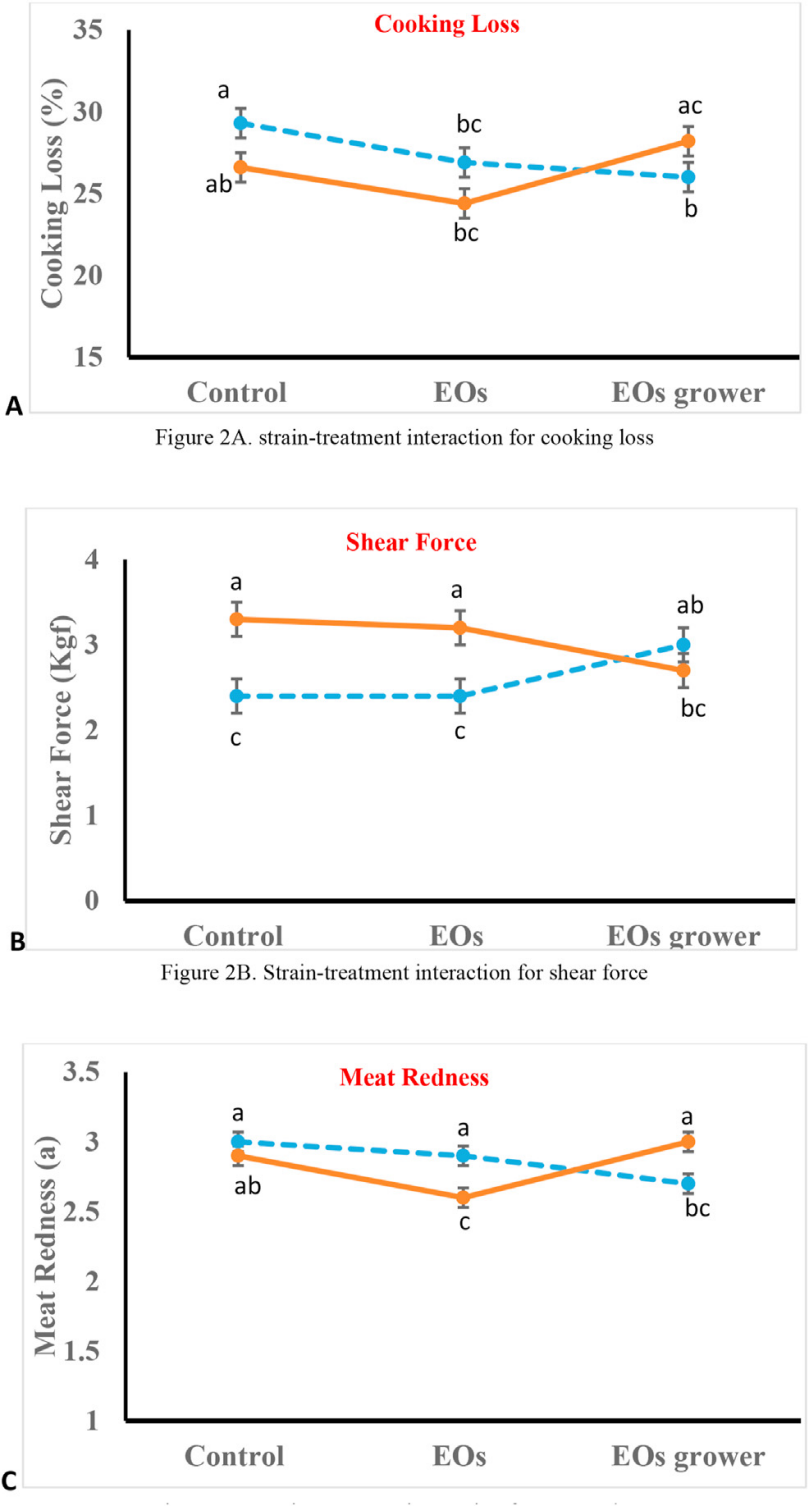
Strain-treatment interaction for meat quality traits ^a, b, c^ Means with different superscripts differ (p < 0.05).

Significant strain by treatment interactions were observed for cooking loss, shear force, and a color coordinate traits indicating that the response for Digestarom inclusion in the diet is different for each strain (Figure 2). The inclusion of EOs has resulted in reducing cooking loos for Hubbard strain compared to the control (*p = 0*.01), while the response in the Indian River for EOs was not statistically significant (Figure 2A). Results for shear force trait are shown in (Figure 2B). inclusion of the EOs in the grower phase has resulted in increasing the shear force value for the Hubbard carcasses, while it has improved the Indian River carcass quality by reducing the shear force values compared to both control and EOs treatment (*p = 0*.002). strain by treatment interactions for color coordinate traits are presented in (Figure 2C). The lowest values for meat redness were observed with the EOs grower treatment for the Hubbard carcasses, while EOs have shown the lowest meat redness value for the Indian River carcasses (*p = 0*.005).

## 4 Conclusion

In general, and based on the current results, Hubbard strain performed better in terms of body weight than Indian River due to their higher feed intake without adversely affecting FCR. At the same carcass weight, birds on essential oils had lower cooking loss indirectly indicating that composition is different for the muscle and fat tissues. Lower cooking loss values were observed with the inclusion of EOs. Essential oils as feed additives can be used as an alternative to antibiotic growth promoters. Further studies are needed to evaluate the efficacy of using essential oils in broiler diet with higher inclusion rate.

## Declarations

### Author contribution statement

Mohammad D. Obeidat: Conceived and designed the experiments; Wrote the paper.

Basheer M. Nusairat: Performed the experiments; Analyzed and interpreted the data.

Belal S. Obeidat: Contributed reagents, materials, Analysis tools or data.

### Funding statement

This research was funded by Deanship of Scientific Research at Jordan University of Science and Technology, grant number (172/2016).

### Data availability statement

Data will be made available on request.

### Declaration of interest’s statement

The authors declare no conflict of interest.

### Additional information

No additional information is available for this paper.

## References

[bib1] Abd El-Hady A.M., El Ashry G.M., El-Ghalid O.A.H. (2020). Effect of natural phytogenic extract herbs on physiological status and carcass traits of broiler chickens. Open J. Anim. Sci..

[bib2] Abdel-Wareth A.A.A., Mobashar M., Shah A., Sadiq A.B. (2022). Jojoba seed oil as feed additive for sustainable broiler meat production under hot climatic conditions. Animals.

[bib3] Abdullah A.Y., Kridli R.T., Shaker M.M., Obeidat M.D. (2010). Investigation of growth and carcass characteristics of pure and crossbred Awassi lambs. Small Rumin. Res..

[bib4] Bedford M.R., Cowieson A.J. (2012). Exogenous enzymes and their effects on intestinal microbiology. Anim. Feed Sci. Technol..

[bib5] Benyi K., Tshilate T.S., Netshipale A.J., Mahlako K.T. (2015). Effects of genotype and sex on the growth performance and carcass characteristics of broiler chickens. Trop. Anim. Health Prod..

[bib6] Bobko M., Hašcík P., Mellen M., Bobková A., Tkácová J., Czako P., Pavelková A., Trembecká L. (2016). Effect of different phytogenic additives on oxidation stability of chicken meat. Potravinarstvo.

[bib7] Bortoluzzi C., Lahaye L., Oxford J., Detzler D., Eyng C., Barbieri N.L., Santin E., Kogut M.H. (2021). Protected organic acid and essential oils for broilers raised under field conditions: intestinal health biomarkers and cecal microbiota. Front. Physiol..

[bib8] Cherian G., Orr A., Burke I.C., Pan W. (2013). Feeding Artemisia annua alters digesta pH and muscle lipid oxidation products in broiler chickens. Poultry Sci..

[bib9] Demir E., Sarica Ş., Özcan M.A., Suiçmez M. (2005). The use of natural feed additives as alternative to an antibiotic growth promoter in broiler diets. Arch. fur Geflugelkd..

[bib10] Dhama K., Latheef S.M.S., Samad H., Karthik K., Tiwari R., Khan R., Alagawany M., Farag M., Alam G.M., Laudadio V., Tufarelli V. (2015). Multiple beneficial applications and modes of action of herbs in poultry health and production-A review. Int. J. Pharmacol..

[bib11] Giannenas I., Tzora A., Bonos E., Sarakatsianos I., Karamoutsios A., Anastasiou I., Skoufos I. (2016). Einfluss von zusätzen von essentiellen Ölen des Oreganos und des Lorbers sowie von attapulgit zum futter auf die chemische zusammensetzung, die oxidationsstabilität, das fettsäuremuster und den mineralstoffgehalt von broiler-brust-und schenkelfleisch. Eur. Poult. Sci..

[bib12] Gibson G.R., Probert H.M., Loo J. Van, Rastall R.A., Roberfroid M.B. (2004). Dietary modulation of the human colonic microbiota: updating the concept of prebiotics. Nutr. Res. Rev..

[bib13] Guan R., Lyu F., Chen X., Ma J., Jiang H., Xiao C. (2013). Meat quality traits of four Chinese indigenous chicken breeds and one commercial broiler stock. J. Zhejiang Univ. - Sci. B.

[bib14] Hazrati S., Rezaeipour V., Asadzadeh S. (2020). Effects of phytogenic feed additives, probiotic and mannan-oligosaccharides on performance, blood metabolites, meat quality, intestinal morphology, and microbial population of Japanese quail. Br. Poultry Sci..

[bib15] Hernández F., Madrid J., García V., Orengo J., Megías M.D. (2004). Influence of two plant extracts on broilers performance, digestibility, and digestive organ size. Poultry Sci..

[bib16] Hristakieva P., Mincheva N., Oblakova M., Lalev M., Ivanova I. (2014). Effect of genotype on production traits in broiler chickens. Slovak J. Anim. Sci.

[bib17] Kalia S., Bharti V.K., Gogoi D., Giri A., Kumar B. (2017). Studies on the growth performance of different broiler strains at high altitude and evaluation of probiotic effect on their survivability. Sci. Rep..

[bib18] Li H.L., Zhao P.Y., Lei Y., Hossain M.M., Kim I.H. (2015). Phytoncide, phytogenic feed additive as an alternative to conventional antibiotics, improved growth performance and decreased excreta gas emission without adverse effect on meat quality in broiler chickens. Livest. Sci..

[bib19] Mahmoud K.Z., Obeidat B.S., Ishmais M.A. (2015). Roasted sesame hulls improve broiler performance without affecting carcass characteristics. Ital. J. Anim. Sci..

[bib20] Mountzouris K.C., Paraskeuas V., Tsirtsikos P., Palamidi I., Steiner T., Schatzmayr G., Fegeros K. (2011). Assessment of a phytogenic feed additive effect on broiler growth performance, nutrient digestibility and caecal microflora composition. Anim. Feed Sci. Technol..

[bib21] Musa H.H., Wu S.L., Zhu C.H., Seri H.I., Zhu G.Q. (2009). The potential benefits of probiotics in animal production and health. J. Anim. Vet. Adv..

[bib22] Peri L., Žiki D., Luki M. (2009). Aplication of alternative promoters in broiler production Probiotics Prebiotics. Biotechnol. Anim. Husb..

[bib23] Pirgozliev V., Mansbridge S.C., Rose S.P., Lillehoj H.S., Bravo D. (2019). Immune modulation, growth performance, and nutrient retention in broiler chickens fed a blend of phytogenic feed additives. Poultry Sci..

[bib24] Ravindran V. (2013). Poultry feed availability and nutrition in developing countries. Poult. Dev. Rev..

[bib25] Rhee K.S., Anderson L.M., Sams A. (2006). Lipid oxidation potential of beef, chicken, and pork. J. Food Sci..

[bib26] Ruff J., Tellez G., Forga A.J., Señas-Cuesta R., Vuong C.N., Greene E.S., Hernandez-Velasco X., Uribe Á.J., Martínez B.C., Angel-Isaza J.A., Dridi S., Maynard C.J., Owens C.M., Hargis B.M., Tellez-Isaias G. (2021). Evaluation of three formulations of essential oils in broiler chickens under cyclic heat stress. Animals.

[bib27] Sadek K.M., Ahmed H.A., Ayoub M., Elsabagh M. (2014). Prüfung von digestarom und thymian als phytogene futterzusatzstoffe bei broilern. Eur. Poult. Sci..

[bib28] Stefanello C., Rosa D.P., Dalmoro Y.K., Segatto A.L., Vieira M.S., Moraes M.L., Santin E. (2020). Protected blend of organic acids and essential oils improves growth performance, nutrient digestibility, and intestinal health of broiler chickens undergoing an intestinal challenge. Front. Vet. Sci..

[bib29] Syed B. (2019). Evaluation of the influence of a phytogenic feed additive on carcass traits in broilers compared to an. Antibio. Growth Promo..

[bib30] Upadhaya S.D., Lee K.Y., Kim I.H. (2016). Effect of protected organic acid blends on growth performance, nutrient digestibility and faecal micro flora in growing pigs. J. Appl. Anim. Res..

[bib31] Windisch W., Schedle K., Plitzner C., Kroismayr A. (2008). Use of phytogenic products as feed additives for swine and poultry. J. Anim. Sci..

